# Toward multiscalar measures of inequality in archaeology

**DOI:** 10.1073/pnas.2400700121

**Published:** 2025-04-14

**Authors:** Enrico R. Crema, Mattia Fochesato, Andrés G. Mejía Ramón, Jessica Munson, Scott G. Ortman

**Affiliations:** ^a^Department of Archaeology, University of Cambridge, Cambridge CB2 3DZ, United Kingdom; ^b^McDonald Institute for Archaeological Research, University of Cambridge, Cambridge CB2 3ER, United Kingdom; ^c^Department of Social and Political Science, Bocconi University, Milan 20136, Italy; ^d^Dondena Centre for Research on Social Dynamics and Public Policy, Bocconi University, Milan 20136, Italy; ^e^Bocconi Institute for Data Science and Analytics, Bocconi University, Milan 20136, Italy; ^f^Centre for Economic Policy Research, London EC1V 0DX, United Kingdom; ^g^Embodied Cognitive Science Unit, Okinawa Institute of Science and Technology Graduate University, Onna-son, Kunigami-gun, Okinawa-ken 904-0412, Japan; ^h^Institut de Ciència i Tecnologia Ambientals, Universitat Autònoma de Barcelona, Cerdanyola del Vallès, Barcelona 08193, Espanya; ^i^Department of Anthropology-Sociology, Lycoming College, Williamsport, PA 17701; ^j^Department of Anthropology, University of Colorado, Boulder, CO 80309-0233

**Keywords:** Gini coefficient, sampling, scale

## Abstract

The Gini coefficient is a statistical measure commonly used to characterize distributions of socioeconomic quantities. Archaeologists and social scientists have recently adopted this method to analyze ancient inequality by targeting specific proxy variables (e.g., residential unit size, burial data, etc.). Variations in the Gini are then examined in relation to key factors such as time, geography, and subsistence. Yet, Gini coefficients could be obtained across different scales of aggregation, from small neighborhoods within a larger settlement to an ensemble of multiple settlements that are part of the same polity. These different scales of aggregation represent considerable methodological and theoretical challenges, as larger scales might, for example, imply greater social and economic variation within groups and thus affect the Gini coefficients. Furthermore, these issues can also be exacerbated by the idiosyncrasies and limitations of historical and archaeological datasets. This paper discusses the potential and challenges of measuring Gini coefficients at and above the scale of individual archaeological sites, contrasting different approaches and discussing how each can reveal insights into different patterns of past wealth inequality, addressing methodological, empirical, and theoretical implications arising from the multiscalar nature of human interactions.

## Significance

Archaeological measures of inequality typically involve using metrics to quantify the extent and shape of the variability of some proxy variable at the site level. Inequality, however, is intrinsically dependent on the social cohort of reference, and as such, metrics are expected to change depending on the scale of aggregation of the proxy variable. Here, we examine the residential unit size disparity of the GINI Project Database across multiple scales of aggregation, introducing statistical methods quantifying inequalities within and between settlements. Our results show how these complementary metrics can provide ways to describe archaeological inequality and estimate sources and magnitudes of sampling bias, providing insights into larger-scale organizational complexities but also the specific challenges posed by such an inquiry.

The sheer number of different measures of inequality developed in the social sciences testify to both its relevance as a subject of study and the methodological challenges involved in reducing the complexity of empirical observations into a pragmatic, comprehensible, and comparable summary statistic (1–3). The intuitive nature of measures such as the Gini coefficient has led to a range of applications in several fields of study, including archaeology (4–7). Notwithstanding the details and the emphasis placed on different methods, the Gini coefficient is fundamentally a statistical measure of dispersion, often designed to characterize distributions that are skewed with long upper tails. Measures of dispersion are, by definition, capturing comparative relationships that can be conceived of as standardized (S) measures of a random variable *x*’s total deviation from an expectation value (E):

[1] $$\text{Inequality}\;\propto \sum_{i}\frac{{\left|x-E\right|}^{q}}{S^{q}}.$$

The variance is just the mean squared difference (q=2) between each observation and the expectation of a population mean, while the Gini coefficient can be defined as the average absolute difference (q=1) of all pairs of observations normalized by the average and divided by two. While other measures of inequality do exist (e.g., Theil index, a measure of the difference between maximum and observed information entropy in the dataset), any such statistic is necessarily based on a clear definition of a social cohort or reference group and a theoretical expectation (e.g., perfect equality).

The definition of a meaningful cohort, however, is not an obvious or simple choice, as social interactions can occur in different ways at different scales. A hamlet of farmers might have relatively low levels of wealth inequality, but when the social cohort of reference includes neighboring villages, larger towns, and cities, levels of inequality can dramatically increase. Increasing the scale of social interaction can offer opportunities for individuals to interact—directly or indirectly—with others possessing larger differences in wealth. It follows that the definition of a meaningful cohort or scale of analysis represents both a challenge and an opportunity. Different scales of analysis can provide different insights into how inequality is structured (see for example 8).

Archaeological and historical quantitative studies on inequality have almost exclusively focused on individual sites or settlements as a primary unit of analysis (but see 9, 10). The choice is inevitably dictated by the nature of the available data, which only occasionally provides a near-complete picture of a small window of past landscapes. While smaller windows can provide insight into local patterns of variation in inequality within settlements (10, see also 11), the extent to which inequality is structured at geographic scales above individual sites is virtually unexplored in archaeology (but see 9). Here, we examine the GINI Project dataset (12) employing a number of different statistical measures (*SI Appendix*, Table S1 for a summary) to characterize how residential disparity—interpreted here as a conservative proxy for wealth inequality [see (12) for detailed discussions on the relationship between our proxy and wealth inequality]—is structured across different spatial scales, and to identify the unique challenges associated with archaeological and historical datasets. The approach we explore is applicable to any two nested levels of aggregation, but here, we specifically focus on individual settlements (defined by the field [site_id] in the GINI Project Database) from 58 regional cultural groups (defined by unique combinations of the fields [Region] and [SitePhase]) that meets our minimum sampling criteria (*Materials and Methods* and *SI Appendix*, Fig. S1). We focus in particular on three key issues: 1) the information gained by quantifying the discrepancies in inequality measures computed at lower and higher levels of aggregation; 2) the impact of sampling error and bias; and 3) the decomposition of inequality measures that account for intersite variations within regional cultural groups.

## Multiscalar Measures of Inequality.

Given two or more scales of aggregation, we can define a minimum of three sets of metrics capturing within-group, between-group, and global levels of inequality, loosely following the ecological concept of α, β, and γ diversity (13). Here, by within-group or α-inequality, we simply refer to measures of inequality computed at the level of the smallest cohort or unit of aggregation. In this paper, we refer to α-inequality as the Gini coefficient of residential unit sizes for each archaeological site. In contrast, we define γ-inequality as the Gini coefficient computed from all residential units within a region during a particular archaeological period. The third form of inequality, β-inequality, refers to the extent and structure of variation in α-inequality within a particular region of interest. In contrast to α and γ-inequalities, a variety of different metrics can be used to characterize the between-group variations in inequality, but in this context, we will calculate β-inequality as the standard deviation of α-inequality within a regional cultural group.

Within-group (α) and global measures (γ) of inequality are expected to be approximately equal in the case, where all residential units are drawn from the same underlying statistical population (but see below). While deviations from such expectations can provide insights into how inequality is structured at different scales of interaction, it is important to remind ourselves that statistical populations are defined by a measure of dispersion (e.g., residential disparity) as well as a measure of scale (e.g., median residential unit size). The two sources of variation have important implications from an inferential standpoint in this context. Discrepancies between α and γ inequalities can be the result of different sites having larger differences in the former (i.e., high β-inequality) but can also be purely the result of differences in median residence size, as a result of, variation in productivity (cf. 14). Indeed, one can envisage a hypothetical situation where all sites have identically sized residential units, leading to low levels of dispersion, but each with a different scale. Under such a scenario, α and β-inequality will be both low, but γ inequality can be potentially large and will be strongly conditioned by the number of samples contributed from each settlement. The difference between γ and the average α inequality, which we here refer to as δ-inequality, thus effectively captures the presence of some within-regional variation, either determined by differences in the inequality between the lowest levels of aggregation (high β-inequality), by differences in the average values observed at such levels, or a combination of the two (see also 15).

An additional challenge is posed by the fact that archaeological data often consist of small sample sizes, and as such potential bias might be encountered in the interplay between the two levels of aggregation (see also 16). Because γ-inequality is, by definition, computed from a larger sample than α-inequality, even if residential units are drawn from the same statistical population, we would expect δ-inequality to be positively biased because of a higher probability of the regional superset to include extreme observations. Simulation analyses (*SI Appendix, Supporting Information Text* and *SI Appendix*, Fig. S2, upper row) confirm that under unbiased random samples, smaller sampling fractions tend to underestimate Gini coefficients due to rare extreme observations not being sampled, i.e., a black-swan effect, with potentially negative implications about the appropriateness of using bootstrapping methods to obtain confidence intervals for Gini coefficients in archaeological datasets (cf. 17).

**Fig. 1.**
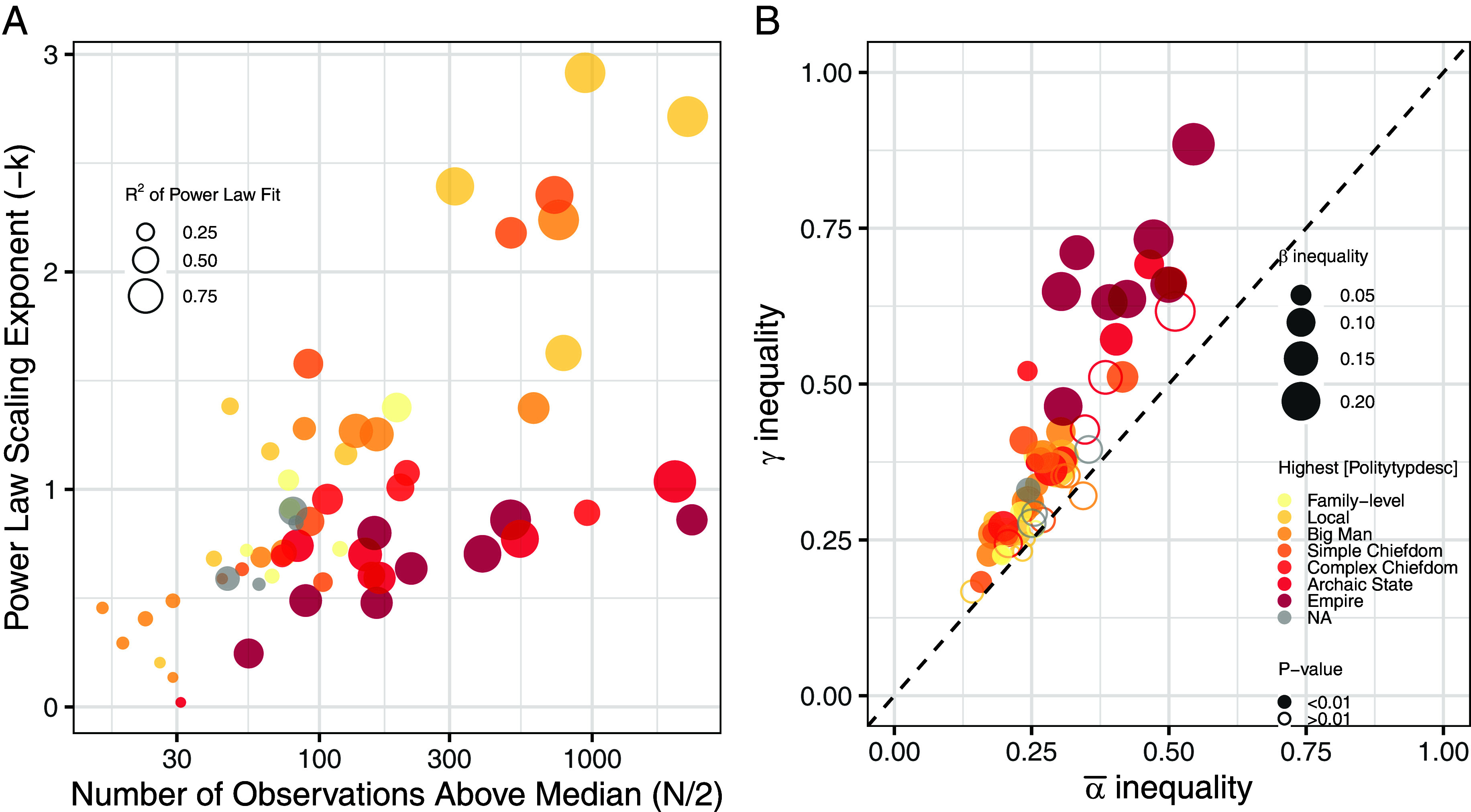
Fit to a power-law distribution and scaling exponent of values of the 58 regional cultural groups meeting the inclusion criteria (*A*); average α, β, and γ inequalities of the same groups, with filled dots showing groups with δ-inequality statistically significant at *P*-value < 0.01 (*B*).

An exploratory log–log power law analysis of the GINI dataset provides further insight into this issue (Fig. 1*A*). Power law distributions emerge when the probability of encountering a given value is proportional to a negative exponent of that value. They often characterize the upper tail of the distribution of wealth in economic systems (18) and are expected with rich-get-richer-type dynamics. A power law distribution of residence size with a decreasing-magnitude scaling exponent $\left(-k\right)$ implies a fatter upper tail and scale invariance that, by extension, results in a greater probability of encountering large values that would tend to increase inequality. The mean is well-defined only when -k≥2. Sampling randomly from a distribution with -k<2 gives a high probability of black swan events, where a new observation might unpredictably and significantly change the expected value. In the case of the GINI dataset, as the number of observations increases, so does the fit of a power law distribution to the upper tail (Log_10_ Logit coeff = 2.014, *P*-value < 0.001) and the magnitude of the scaling exponent (coeff = 0.383, *P* < 0.001), suggesting scale invariance along the tails and that ‘rich get richer’ dynamics are characteristic of past societies. With the exception of six societies (E. Asia—Jomon, E. Asia—Yayoi, E. Asia—Mumun, and SE Europe—Late Neolithic, E Europe Tripolye B1, E Europe Tripolye C1), all have values of -k<2, suggesting a high probability of ‘black swans’.

The potential presence of black swans in the GINI Database effectively implies that we should expect to observe positive values of δ-inequality even in case we do not observe significant intersite variations in residential disparity and size. To account for black swan effects and to determine whether empirically observed δ-inequalities are different from one expected when residential sizes are drawn from the same statistical population, we carried out a permutation test by randomly reassigning each residential unit to a different site in the same regional cultural group.

Fig. 1*B* shows the result of such analyses, along with the β and γ, and average α inequalities. The permutation test shows that 42 out of the 58 unique combinations of [Region] and [SitePhase] have higher than expected values of δ-inequality, based on 1,000 permutations and a significance level of 0.01. Perhaps unsurprisingly, our analyses suggest that small-scale societies generally exhibit low values of α, β, γ, and δ inequalities indicating both low and similar levels of residential disparity and little to no variation in average sizes. In contrast, more complex polities, such as empires and archaic states, have high levels of all metrics, suggesting not just an increase in residential disparity at the site level but also in how such disparities are distributed and structured within a region (*SI Appendix*, Fig. S3). Indeed, the difference between γ and the average α inequality (i.e., δ-inequality) is strongly correlated to the number of levels observed in settlement hierarchy ([NOfLevels], spearman’s rho = 0.47, S = 17286, *P*-value < 0.0001). Furthermore, groups with higher δ-inequality exhibit a positive correlation with β-inequality (Pearson’s r = 0.61, 95% CI: 0.42 to 0.75, t = 5.7552, d.f. = 56, *P*-value < 0.0001) and the standard deviation of mean log residential size (Pearson’s r = 0.81, 95% CI: 0.70 to 0.88, t = 10.352, d.f. = 56, *P*-value < 0.0001), suggesting both intersettlement variation in residential disparity and size as contributing factors.

The latter poses important interpretative issues. On the one hand, if we assume that the relationship between wealth or income and residential units is stationary across settlements within a regional cultural group, the correlation between δ-inequality and the standard deviation of mean log residential size could be interpreted as variation in productivity. On the other hand, if such a relationship varies across settlements due to the presence of confounders, the discrepancy between γ and the average α inequality might reflect the intersite variation in those confounders. For example, in contemporary industrial societies, the relationship between residential size and income or wealth might be different between large cities and small regional towns due to differences in average house prices per square meter. The issue is further exacerbated by the fact that large cities and smaller regional towns might also exhibit differences in inequalities, with the former typically showing higher values (11, 14, 19).

## Sample Size Effects.

While measures of δ-inequality can potentially reveal the presence of heterogeneity in the structure of residential disparity, the extent to which γ-inequality can be interpreted as a measure of global inequality is questionable, especially considering the nature of the archaeological record. First, while complete samples are rare, site-level α-inequality can be considered sufficiently robust for many archaeological sites that have undergone extensive excavation or geophysical investigations. In contrast, the sampling fraction for regional systems is likely going to be small. Second, in both settlement and regional contexts, archaeological sampling might be biased toward the selection of larger residential units (for the former) and larger settlements (for the latter) due to research bias and archaeological visibility. This size bias can cause a reverse black swan effect, and under a small sampling fraction and high disparity, it could lead to an overestimate of the Gini coefficient (*SI Appendix*, Fig. S2 lower row).

The result of this potential bias can, in part, explain the patterns observed in Fig. 1*A*. All societies with fewer than 1,000 observations and all complex societies are associated with a scaling exponent smaller than -k<2, which would imply a distribution with a poorly defined mean. While the mean might be overly sensitive to extreme values, a more likely explanation is that large sites and residential units have been oversampled, resulting in a less steep *−k,* and a poor fit due to insufficiently sampled small residential units. This suggests that inferences about residential disparity in regions with -k<2 are unreliable, given that the most representative households are heavily undersampled.

The implications of black swan and reverse black swan effects are further exacerbated by the potential effect of population size on inequality. As mentioned above, in contemporary industrial societies, we often observe a positive correlation between city size and income inequality (20), and similarly, archaeological and historical samples show higher levels of residential disparity in larger urban centers compared to smaller settlements (21–23). It follows that regional residential disparity (i.e., γ-inequality) is conditioned by the hierarchy in settlement size, its potential relation to inequality, and the potential presence of black swan and reverse black swan effects.

Here, we approach the issue by testing the method and insights presented in (9) on the GINI database. There, the authors dealt with the issue of population effect on inequality in an archaeological dataset by estimating a measure called the scale effect, here referred to as simply η, which examines α- and γ-inequality accounting for the population sizes at different scales of aggregation. As regions and sites within regions can be assumed to be similar in other factors (politics, technologies, demographics, and culture) that might influence (both directly and indirectly) the effect of population on inequality, *η* has the property of capturing the effect of population on inequality that depends only on population scale. More formally, we take our sample set of unique combinations of [Region] and [SitePhase] and compute our statistic *η_ij_* for each settlement *i* in the regional cultural group *j* as follows:

[2] ηij=(Gj-Gi)/(nj-ni),

where *G_i_* is the Gini coefficient (α-inequality) of site *i*, *G_j_* is the Gini coefficient of the region (γ-inequality), and *n_i_* and *n_j_* are the number of samples at the site and regional level. With all things being equal (e.g., variation in median residential size), systems characterized by small β-inequality (i.e., low intersite variation in α-inequality) are expected to consistently yield an *η_ij_* close to zero. Positive values of *η_ij_* indicate that the focal site is characterized by α-inequality < γ-inequality, while negative values suggest the opposite, with the absolute deviation from 0 being dictated by the relative (sample) size of the focal community.

**Fig. 2.**
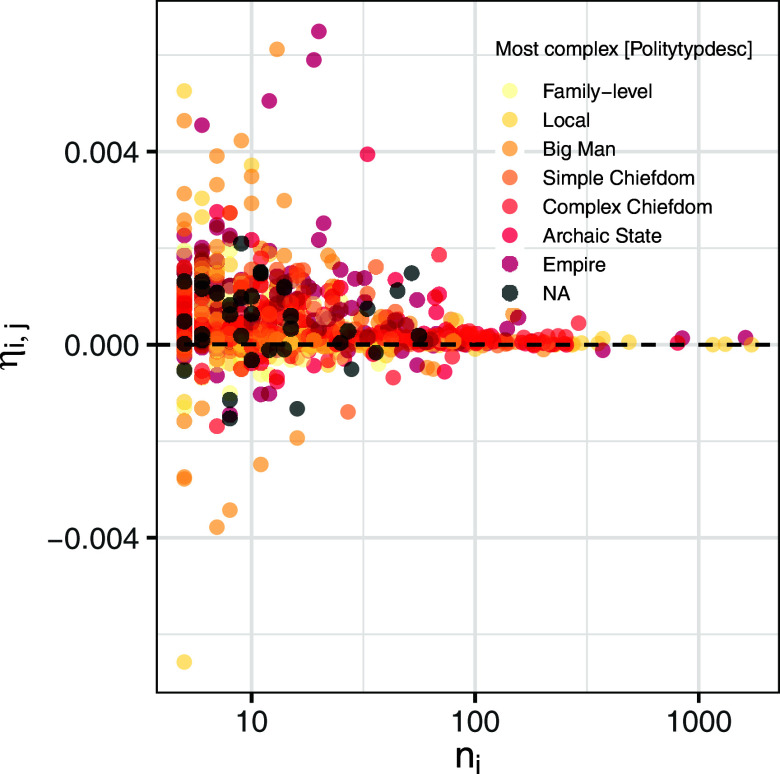
Scale effect *η_ij_* and number of residential units (*n_i_*) the subset of 1,018 sites from the GINI Database.

Residential data from the GINI database show (Fig. 2), across all polity levels, a heteroskedastic pattern with *η_ij_* showing high positive and negative deviations from 0 at lower sample sizes. With larger values of *n_i_*, typically over the threshold of one hundred units, *η_ij_* consistently tends toward zero, indicating that across our sample, comparatively larger settlements exhibit α*-*inequalities closer to γ*-*inequality. The standard deviation of *η_ij_* within each cultural region does not show any significant correlation with [NOfLevels] (Spearman’s rho = 0.06, S = 30,511, *P*-value = 0.65), indicating that the variability of the scale effect is not conditioned by the presence of settlement hierarchy. No consistent patterns are observed in relation to polity levels either (*SI Appendix*, Fig. S4), with a considerable variation of the scale effect observed, for example, for big man polities and archaic states, while empires do not exhibit a similarly large variation of *η_ij_*. The variability of *η_ij_* seems to be instead primarily determined by the overall sample size, with a negative correlation between its standard deviation and the total number of residential units within a region (Pearson’s r = −0.39, 95% CI: −0.59 ~ −0.15, t = −3.19, df = 56, *P*-value = 0.002). This confirms that the scale effect captures only the sample size effect on inequality across units that are similar in other aspects and possibly controls for the effect of political factors on the impact of sample size on inequality. Our results suggest that the magnitude of the pure population effect on inequality depends mostly on the sample size. The smaller the sample size of a site, the larger the effect of scale on inequality when comparing the site to regional Gini coefficients, with the numerator of equation [2] consistently yielding positive values (i.e., γ-inequality > α-inequality) due to the black-swan effect.

**Fig. 3.**
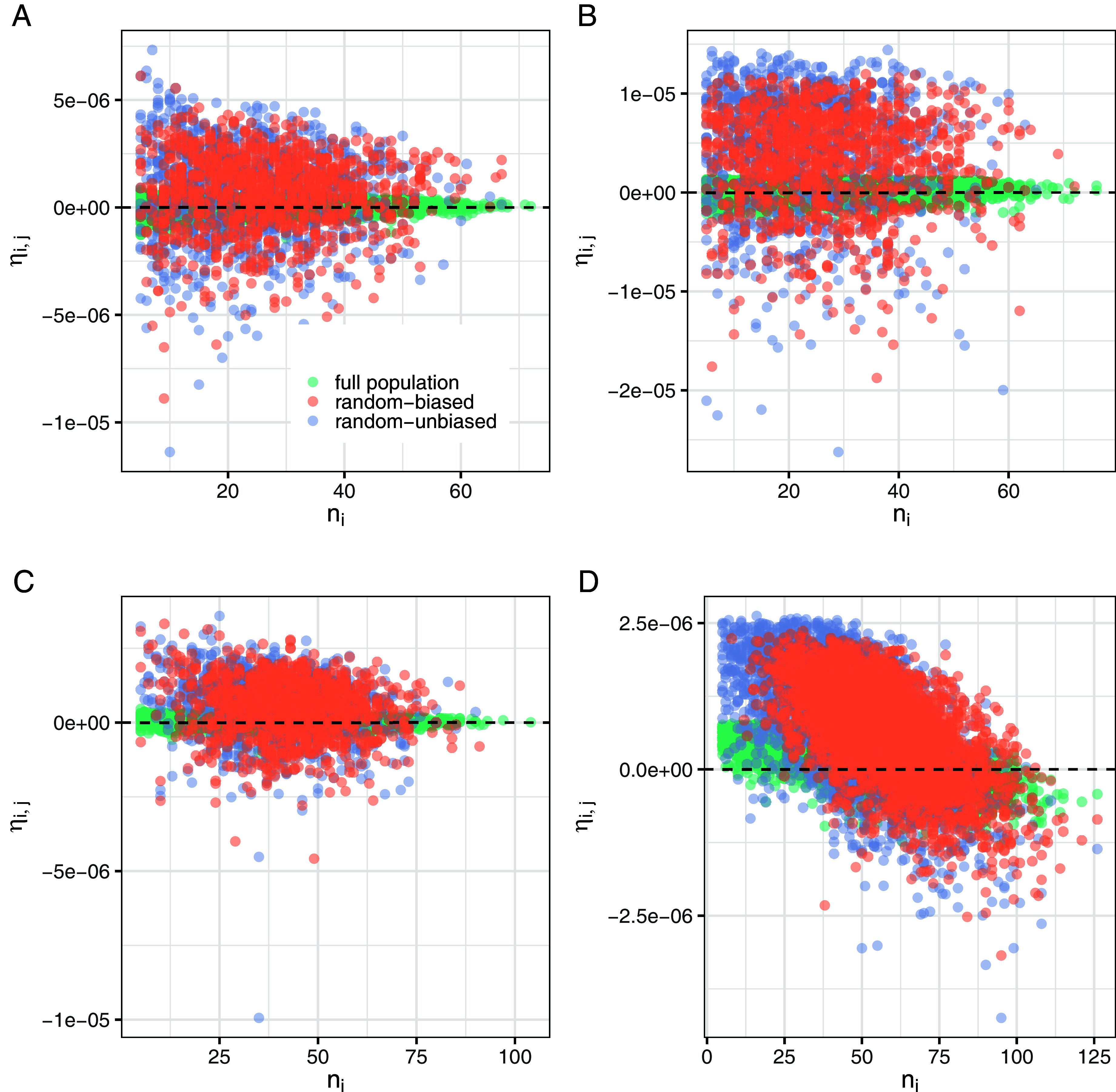
Scale effect *η_ij_* vs sample size *n_i_* computed on complete and sampled (biased and unbiased) simulated datasets: (*A*) low β inequality and no correlation between α inequality and *n_i_*. (*B*) high β inequality and no correlation between α inequality and *n_i_*. (*C*) low β inequality and positive correlation between α inequality and *n_i_*. (*D*) high β inequality and positive correlation between α inequality and *n_i_*.

The results presented above are conditional on the assumption of a comparatively high sampling fraction at both the regional and site level. While this assumption can be held in some historical contexts, the vast majority of archaeological datasets are characterized by small, and potentially biased samples, potentially characterized by regional-scale reverse black-swan effect (i.e., larger settlements having a higher chance of being sampled and excavated). Fig. 3 explores how *η_ij_* is conditioned by small sampling fractions and nonrandom selection of archaeological sites using simulated datasets under a variety of settings. In all cases, *η_ij_* is expected to be close to zero unless we have high β-inequality and a positive correlation between α-inequality and *n_i_*. Under this scenario (Fig. 3*D*), we expect to observe a negative correlation between *η_ij_* and *n_i_*. In all instances, random samples generally tend to have a higher variance in *η_ij_* with the effect slightly more pronounced with lower values of *n_i_*. A slightly different scenario is again observed when residential disparity and size are correlated. Under such a setting, the slope of the relationship between *η_ij_* and *n_i_* becomes steeper for samples compared to the full population under both biased and unbiased sampling regimes.

When examined along with the observed data, the lack of correlation between scale effect and sample size suggests that we should not observe a strong correlation between residential disparity and sample size, a pattern confirmed by the lack of a statistically significant correlation between α-inequality and *n_i_* (Pearson’s r = 0.002, 95% CI: −0.04 ~ 0.08, t = 0.729118, d.f. = 1,016, *P*-value = 0.4661). More generally, this exercise shows that the pure scale effect of population on inequality is small, and it is likely to be smaller in reality (complete datasets) than the one computed on sampled sets of sites, but the process of sampling matters. Small and/or biased samples, which overrepresent larger sites, might substantially overestimate the computation of the absolute scale effect at low population levels.

## Disentangling Inequalities.

While contrasting γ and α inequalities directly can reveal the presence of heterogeneity in how inequalities are structured within a region, a more robust alternative requires the decomposition of each observation’s contribution to the total regional pattern. Such a process allows the disentangling of the in-group and out-group inequalities and their statistical significance given the distribution of values within the dataset. In our case, the in-group inequality contrasts each residential unit to the size of all other units in the same settlement, while the out-group inequality contrasts each residential unit with those of other settlements in the same region. The decompositions rely on the fact that inequality is a standardized deviation from an expectation and the assumption that total inequality is a weighted sum of the in-group and out-group inequalities experienced by each observation. More specifically, by combining these in-group and out-group Inoua inequalities (24), it is possible to derive the total inequality experienced by each observation, and by computing the total weighted sum, the total inequality of a region. A key advantage of this approach, detailed in (25), is the intuitive appeal of formalizing the concept of inequality as a comparative measure, where one can define the relational structure (e.g., it is possible to employ a probabilistic measure of membership to a group) and the expectation (e.g., it is possible to use alternatives to sample means).

Statistical significance of observed patterns can be derived by contrasting this to expected statistics derived from randomly permuted group memberships. Thus, an observation experiences statistically significant in-group inequality relative to its society if its in-group component is greater (or lower, for significant equality) than the out-group component, and if this difference is greater than the average difference when group and out-group members are randomized. Likewise, an observation experiences significant out-group inequality relative to its society if its out-group component is less than the total inequality experienced by all observations—and this value is more extreme than the equivalent difference when group and nongroup members are randomized—when the nongroup excludes comparisons to self or its group (either directly, or via the global mean) given that excluding the observation’s group will tend to make the rest of society look more equitable. The converse is true if the out-group comparison is limited to the out-group mean and/or the value of each observation itself. Similar inferences can be made from the weighted sum of all permuted values for whether a regional cultural group has higher in-group and out-group inequality than would be expected compared to a null of all groups being drawn from the same distribution.

**Fig. 4.**
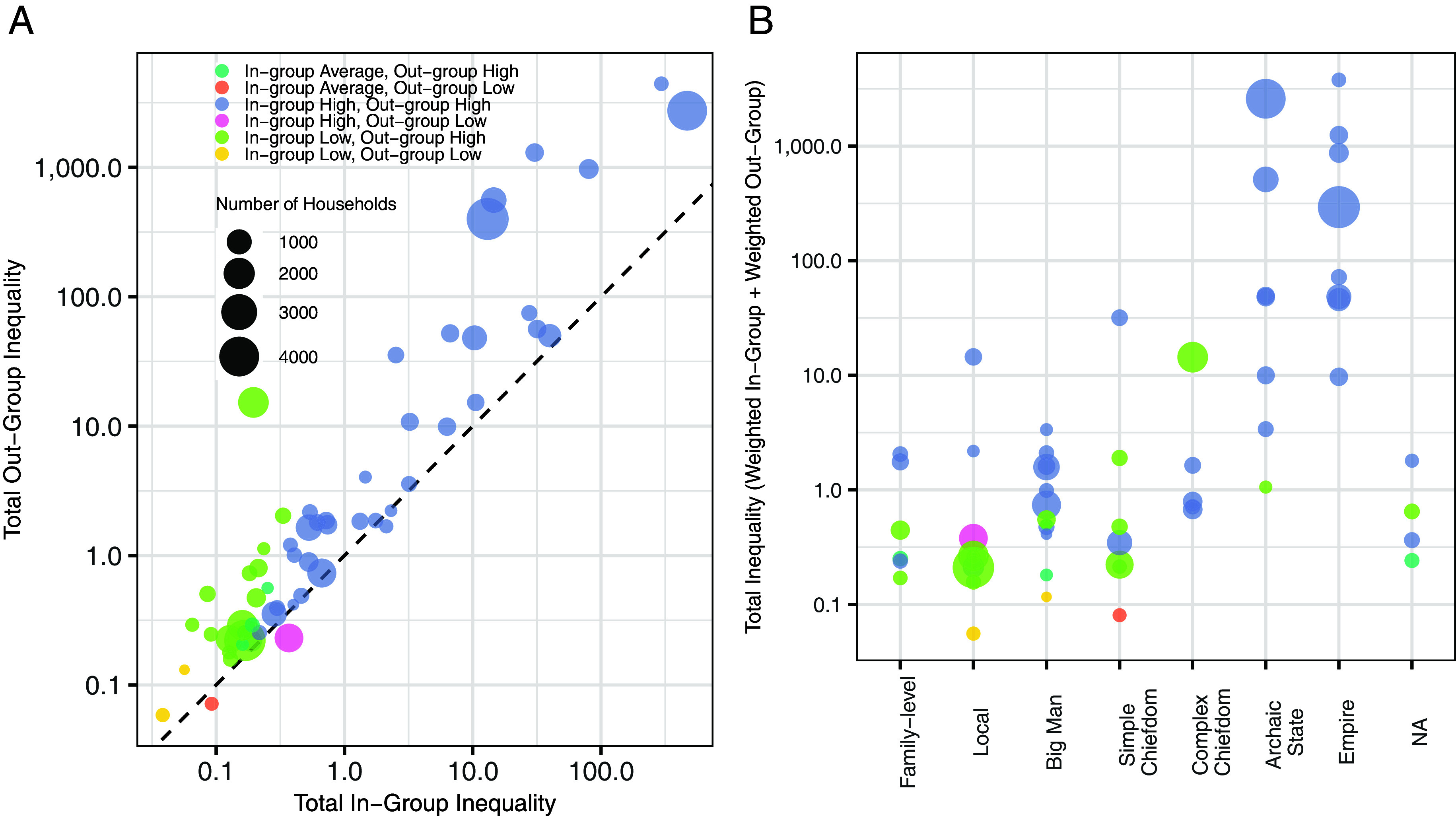
(*A*) Global within-group vs. across-group inequality for the 58 regions. (*B*) Total inequality by organizational complexity.

Fig. 4 shows such inferences summarized at the level of each region, with the site (or nonsite) mean as the expectation and each observation’s value as the standard. So, for each observation *i*, *i* ‘judges’ all other observations *j*, expecting them to be equal to the site (or nonsite) mean. *i* then determines how meaningful this difference to the mean is to *j* as a fraction (or multiplier) of *j*’s wealth. As in-group inequality increases, so does out-group inequality (Fig. 4*A*; log–log coef = 1.18, *P* < 0.001), reproducing, in a complementary way, the findings from Fig. 1*B*. In all but three instances, in fact, we observe total out-group inequality to be larger than the in-group inequality, suggesting that observations typically encounter more inequality when comparisons are made to members outside its group. The analyses also show a clear association between total inequality and polity level, with polities below the level of state showing often lower than-expected in-group inequality. Moreover, the total inequality of states and empires is greater than the total inequality of chiefdoms and less-complex societies (one-sided Wilcoxon rank sum exact test, W = 61, *P* < 0.0001), with total inequality also positively correlated to [NOfLevels] (spearman’s rho = 0.56, S = 14,168, *P*-value < 0.0001). With the exception of S Africa—Zimbabwe and Southern Andes—Late Intermediate, all regions that can be said to have area segregation by site (a Low in-group/High out-group inequality inference, based on a permutation test with 100 iterations) are all Chiefdoms or simpler (Fig. 4*B*), demonstrating how the structure of intra- and intergroup inequality changes with organizational complexity. In all but four cases (E Europe—Tripolye B; Northwest NA—Early Koniag; Northwest NA—Early Pacific; W Europe—Late Bronze Age), we observe higher levels of out-group inequality than would be expected if households were randomly distributed within society, suggesting that despite differences in segregation, residence areas are nevertheless autocorrelated by settlement.

However, as shown by Supplemental *SI Appendix*, Figs. S5–S12, inferences at the regional level are not always representative of inferences at the local level. For example, Mumum East Asia (S5) has a number of observations with significantly higher in-group inequality than would be expected due to chance, but the large number of sites with significantly low inequality and the low variance of the in-group inequality results in a global inference of lower-than-expected in-group inequality. Likewise, only a handful of values in Iron Age West Asia and Cyprus (S9) have significantly higher-than-expected in-group inequality, but the large variance of in-group values nevertheless results in higher-than-expected in-group inequality on the highest level of aggregation.

## Summary.

The methodological overview and the quantitative assessment of the GINI dataset presented here highlight the unrepresentativeness of inequalities calculated exclusively at the level of each site (i.e., α-inequality) and the inadequacy and limitations of inequalities calculated by assembling all observations within a larger social cohort (γ-inequality). In the case of residential disparity, α-inequality can rely on a higher sampling fraction and a more consistent relationship between the proxy and the target variable. However, the scale of a settlement underrepresents the range of social interactions experienced by individuals, and the extent by which inequality metrics calculated at this scale are representative of entire cultures is conditioned by the degree of variation between settlements. Indeed, spatial variation in income and wealth inequalities is consistently observed in modern nation-states (19, 26, 27), and a key question is to determine when and how such patterns emerged in human history, and what methodological challenges one needs to account for.

Societal-level calculations of inequality (γ-inequality) are, in this sense, limited by a number of factors. Although they provide larger sample sizes and capture the widest range of social interactions, they do not capture the level of heterogeneity in inequality across sites nor the fact that the relation between wealth or income and residential floor area per se might be different in different communities. With increasing scales confounding factors are likely to play a bigger role in observed signatures. In the case of residential disparity, variations in demography, economic organization, and dwelling types can all confound the relationship to housing inequality, and not necessarily in predictable ways. The problem is further exacerbated by the fact that a meaningful unit of aggregation is harder to define in archaeological contexts, particularly in smaller-scale societies. In this sense, our attempt to use the combination of fields [Region] and [SitePhase] has likely led to a wider social cohort than the one experienced by individuals, which, in turn, might explain why we consistently observe significant out-group inequality across our dataset. This potential bias is undoubtedly further conditioned by issues of chronological resolution and time-averaging. Finally, potential associations between inequality and population size can also be a confounder, and even in the absence of these factors, one would still need to deal with small sampling fractions, and the potentially nonrandom nature of the sampling process due to the idiosyncrasies of archaeological research. While the impact of these issues varies depending on the specific proxy being examined, it is unquestionable that possibilities offered by examining multiple scales is not a free upgrade and comes with the cost of a more tangled relationship between measured variables and the type of inequality of interest.

Despite these shortcomings, our analyses show that contrasting the two scales of analyses, whether taking just the difference between γ-inequality and average α-inequality, computing site-level statistics that account for differences in population sizes across scales, or distinguishing between in-group and out-group inequalities, can all provide important insights at the level of each residential unit, site, and region that are not possible when using standard measures of inequality computed in archaeological datasets. Although our analyses were limited to a subset of 58 unique regional cultural groups, our exploration consistently showed a correlation between polity levels and settlement hierarchy on the one hand and metrics capturing how residential disparity is structured within a larger aggregation unit, providing a much more nuanced picture than what can be offered by a single axis of variation based on site-level Gini coefficients.

## Materials and Methods

### Material.

We analyzed a subset of the GINI dataset considering two scales of analyses: site and regional cultural group. The former is based on the [site_id] field, while the latter was derived based on unique combinations of the fields [Region] and [SitePhase]. We filtered the GINI dataset considering only sites with five or more residential units and regions with five or more sites. Our final dataset consisted of 35,641 observations from 1,018 sites from 58 regional cultural groups. Variables recorded at the site level, such as [TypeOfPolity] and [NOfLevels], were summarized at the largest recorded value. All inequality calculations were based on the field [TotalAreaHouse].

### Methods.

#### Simulation of artificial archaeological datasets.

Simulated values of residential sizes were obtained by sampling from a Fisk distribution (28), which has a scale parameter equal to the median and a shape parameter which is equal to the reciprocal of the Gini coefficient. We generated four datasets, each with 10,000 sites using a scale parameter equal to 20 and a shape parameter randomly sampled from a beta distribution with a mode of 0.2 and a concentration of 200 (Fig. 3 *A* and *C*, low β-inequality) or 10 (Fig. 3 *B* and *D*, high β-inequality). The number of residential units for each site was either drawn randomly from a rounded and truncated normal distribution with a mean of 20 and a standard deviation of 15 (Fig. 3 *A* and *B*) or by sampling from the same model but with a mean equivalent to the sampled Gini coefficient multiplied by 100 to emulate instances of larger settlements having higher α-inequality. A subsample of 1,000 sites was obtained from the full set, with the probability of each site being selected based on the following equation:

[3] $$\pi_{i}=\frac{s_{i}^{b}}{\sum_{j=1}^{n}s_{j}^{b}},$$

where π_i_ is the probability of sampling the site *i*, *s_i_* is the size of *i*, and *b* is bias coefficient, equal to 0 for an unbiased sample, and positive (b = 0.5 in our case) for a bias favoring the selection of larger settlements.

#### Inequality decomposition via local indicators of dispersion.

Given a weights matrix ***W*** with elements $w_{\mathrm{ij}}$ representing the probability that *j* is a group (G) member to *i*, the probability that *j* is not a member of *i*’s group (NG) is 1 − *W*. Thus, the in-group inequality $J_{\mathrm{Gi}}$ experienced by observation *i* is

[4] $$J_{\mathrm{Gi}}=\sum_{j=1}^{n}\frac{w_{\mathrm{ij}}{\left|x_{j}-E(x_{j})\right|}^{q}}{2\sum_{j=1}^{n}\left[w_{\mathrm{ij}}\right]S(x_{i}{)}^{q}}.$$

Whenever *j* is definitely a member of *i*’s group, $w_{\mathrm{ij}}=1$ in the numerator and *j’*s standardized deviation to the expectation fully contributes to *i*’s perceived within-group inequality. Whenever *j* is definitely not a member of *i’*s group, $w_{\mathrm{ij}}=0$ and *j* is excluded. The row-sum of the weights matrix in the denominator sums to the total number of members in *i*’s group, and ensures that the total $J_{\mathrm{NGi}}$ is a weighted sum. The factor of $\frac{1}{2}$ is retained for historical reasons by analogy to the Gini index. The out-group inequality $J_{\mathrm{NG}}$ as experienced by J is simply

[5] $$J_{\mathrm{NGi}}=\sum_{j=1}^{n}\frac{(1-w_{\mathrm{ij}}){\left|x_{j}-E(x_{j})\right|}^{q}}{2(n-\sum_{j=1}^{n}w_{\mathrm{ij}})S(x_{i})},$$

where the term $1-w_{\mathrm{ij}}$ in the numerator selects for nongroup members, and the second term in the denominator counts the total number of observations not in *i*’s group. Following from the second assumption, the total inequality experienced by *i* is the sum of the group and nongroup components weighted by the number of group- and nongroup members:

[6] $$J_{i}=J_{\mathrm{Gi}}\sum_{j=1}^{n}\frac{w_{\mathrm{ij}}}{n}+J_{\mathrm{NGi}}\sum_{j=1}^{n}\left[1-\frac{w_{\mathrm{ij}}}{n}\right],$$

and the total inequality of the dataset *J* is the weighted sum of all inequalities perceived by all observations:

[7] $$J=\sum_{i=1}^{n}\sum_{j=1}^{n}J_{\mathrm{Gi}}\frac{w_{\mathrm{ij}}}{n^{2}}+J_{\mathrm{NGi}}\left(1-\frac{w_{\mathrm{ij}}}{n^{2}}\right).$$

Here, we take q=2 following (24) due to its tail sensitivity the expectation E as the (non)-site mean value. $\sum_{j=0}^{n}w_{\mathrm{ij}}x_{j}$ and the standard S to *j*’s value $x_{j}$.

## Supplementary Material

Appendix 01 (PDF)

## Data Availability

All scripts and data for replicating the analyses and reproducing main and supplementary figures are provided as an R script (29).
