# Supplementary material for: Toward multiscalar measures of inequality in archaeology
Source: Proc Natl Acad Sci U S A. 2025 Apr 14;122(16):e2400700121. doi: 10.1073/pnas.2400700121 (PMC12037059; doi:10.1073/pnas.2400700121)
Supplement: Supplementary file 1 — Appendix 01 (PDF) [file pnas.2400700121.sapp.pdf]

PNAS

Supporting Information for
Towards Multiscalar Measures of Inequality in Archaeology

Enrico R. Crema
Mattia Fochesato
Andrés G. Mejía Ramón
Jessica Munson
Scott G. Ortman

Enrico R. Crema
Email: erc62@cam.ac.uk

This PDF file includes:

Figures S1 to S12
Table S1

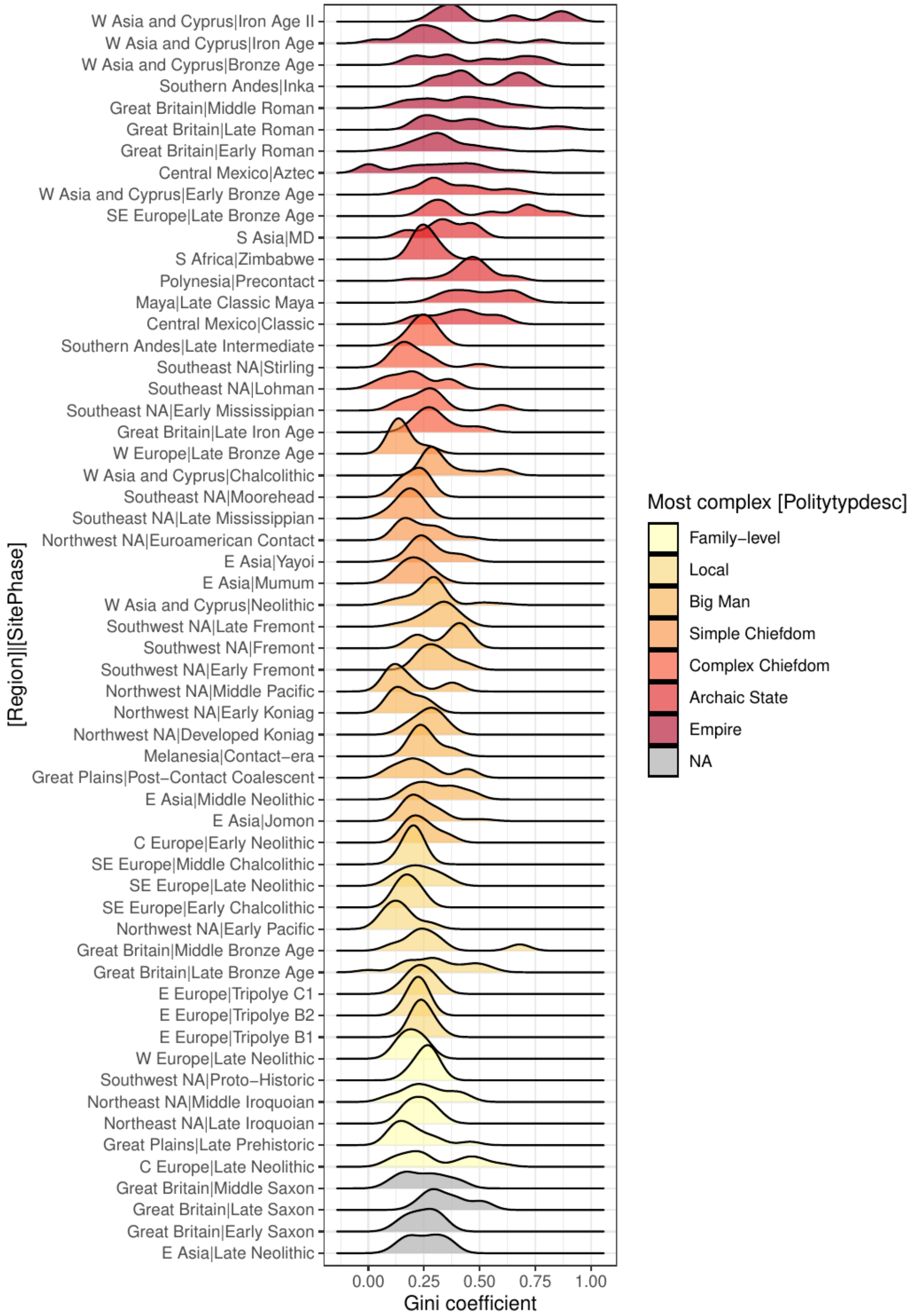

Fig. S1. Distribution of site-level Gini coefficients across different combinations of [Region][SitePhase].

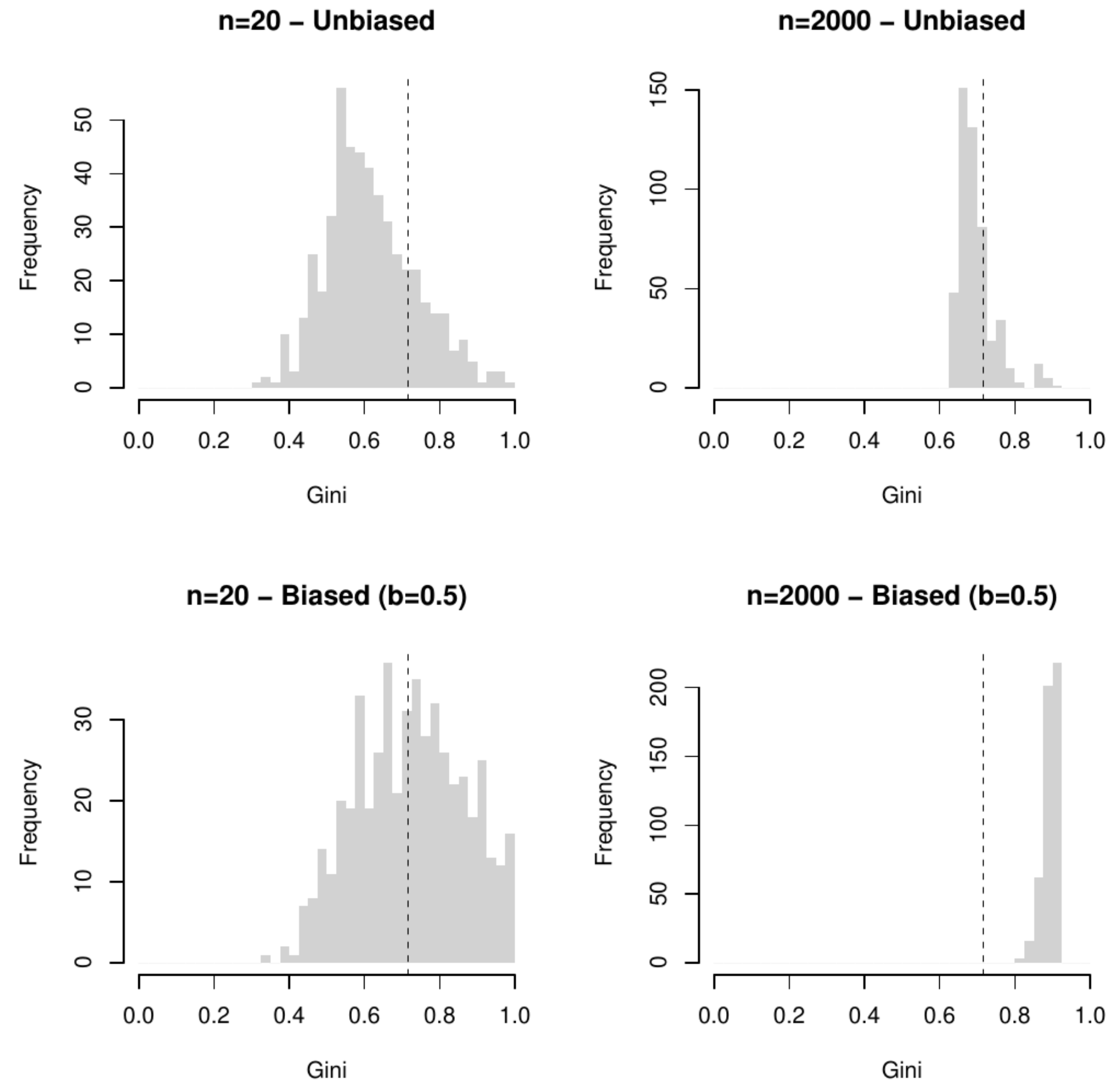

Fig. S2. Impact of sampling fraction and bias in the calculation of Gini coefficients. Each panel shows the distribution of 500 Gini coefficients calculated on a random or biased (with b=0.5, see *Materials and Methods* in the main text) sub-sample of size *n* from a population of 100,000 simulated measurements. The dashed vertical line represents the 'true' Gini coefficient measured at the population level.

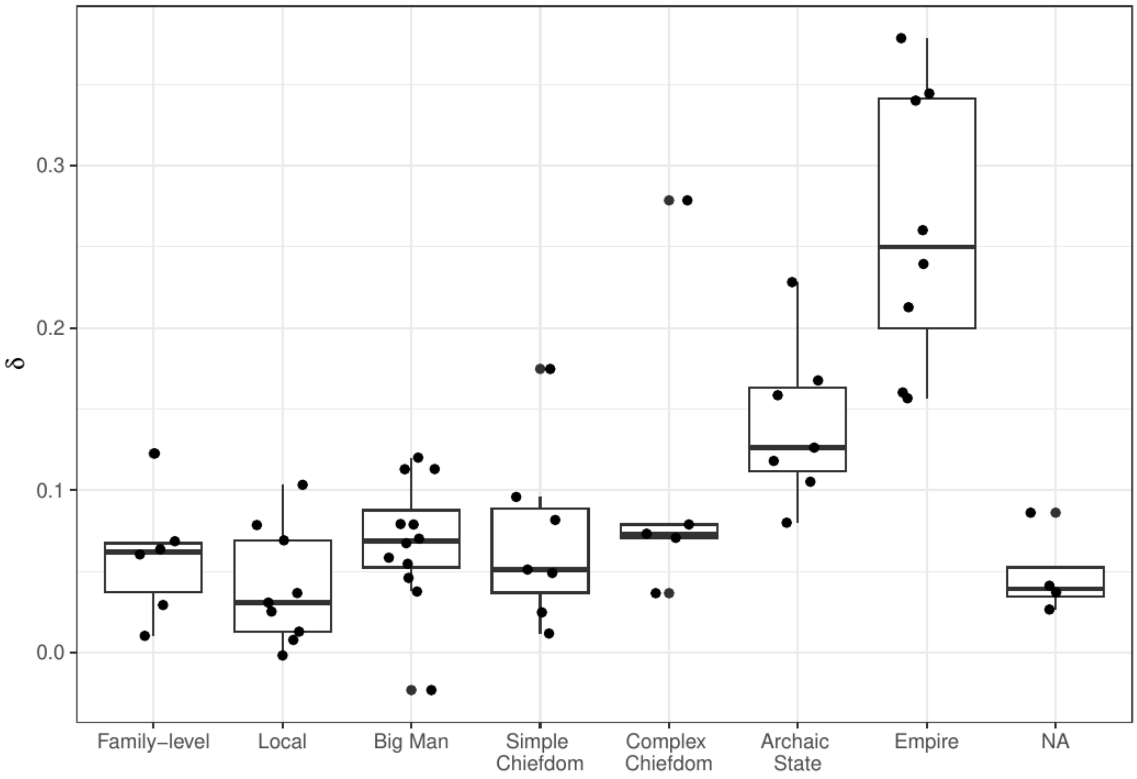

Fig. S3. δ-inequalities measured on the sample of 58 [Region][SitePhase] examined.

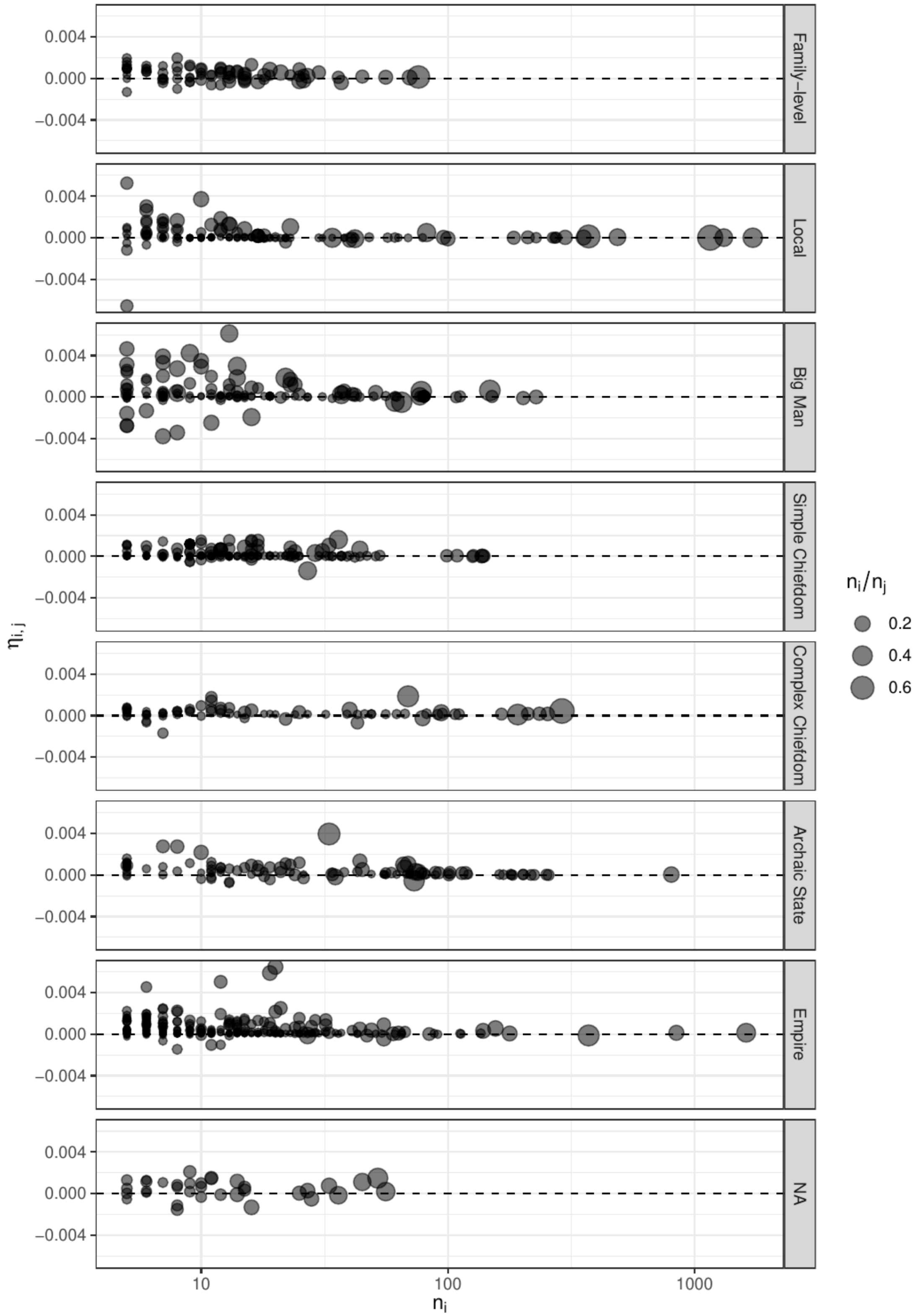

Fig. S4. Scale effect (η_{ij}) and number of residential units (n_i) on a sample of 1,018 archaeological sites organised by polity level.

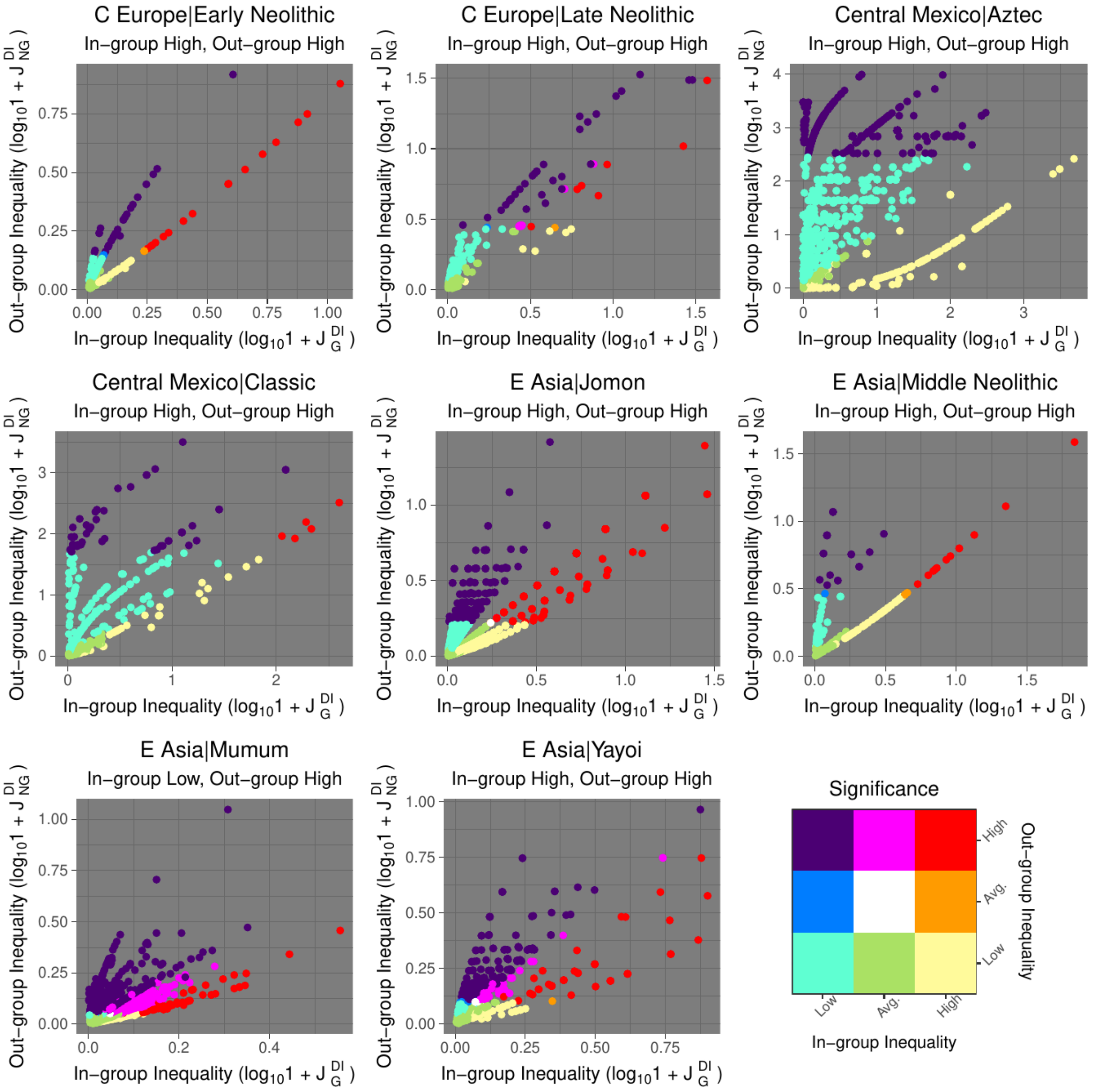

Fig. S5. Residential unit level in-group and out-group inequality per [Region][SitePhase] (1 of 8)

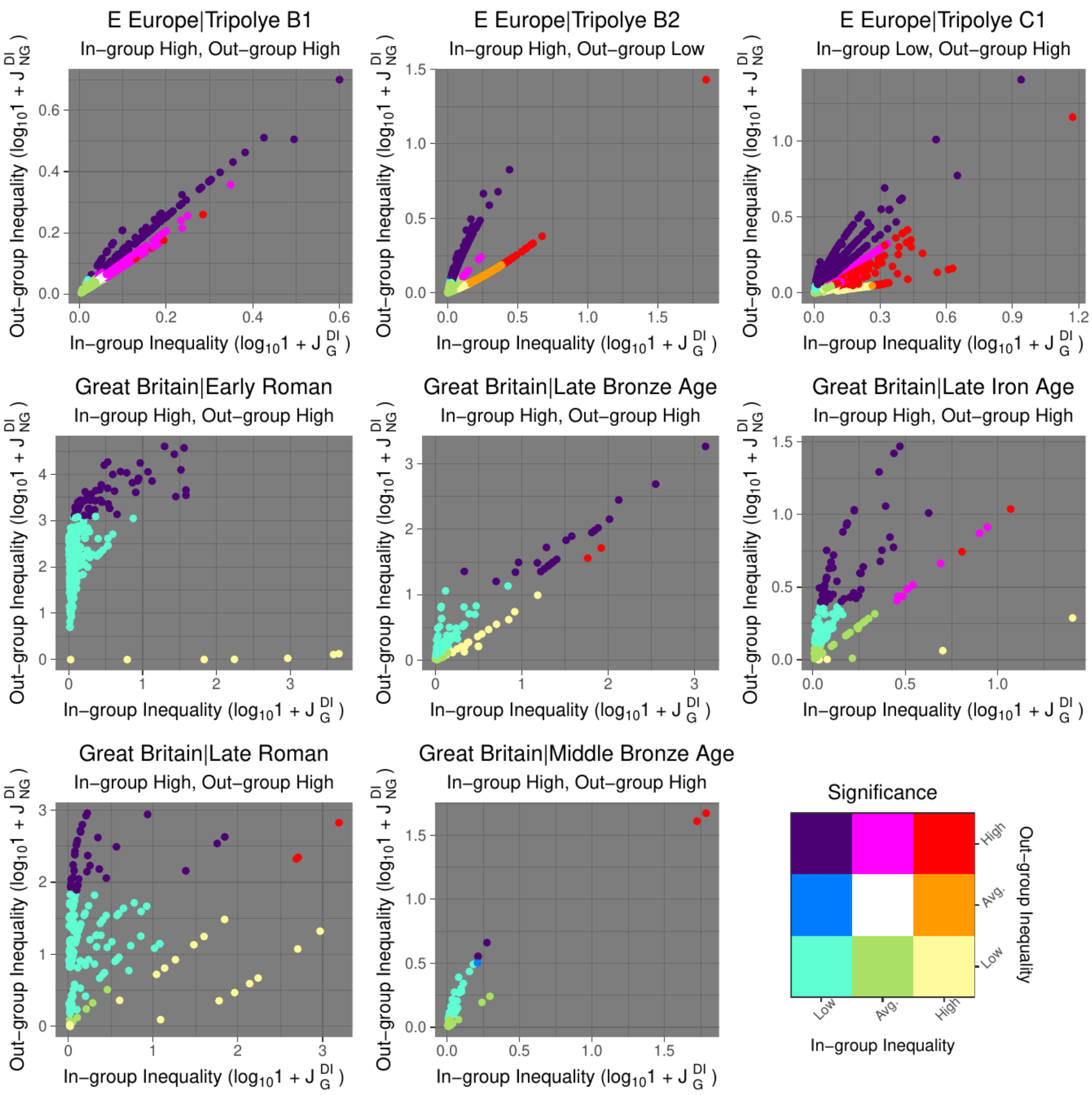

Fig. S6. Residential unit level in-group and out-group inequality per [Region][SitePhase] (2 of 8)

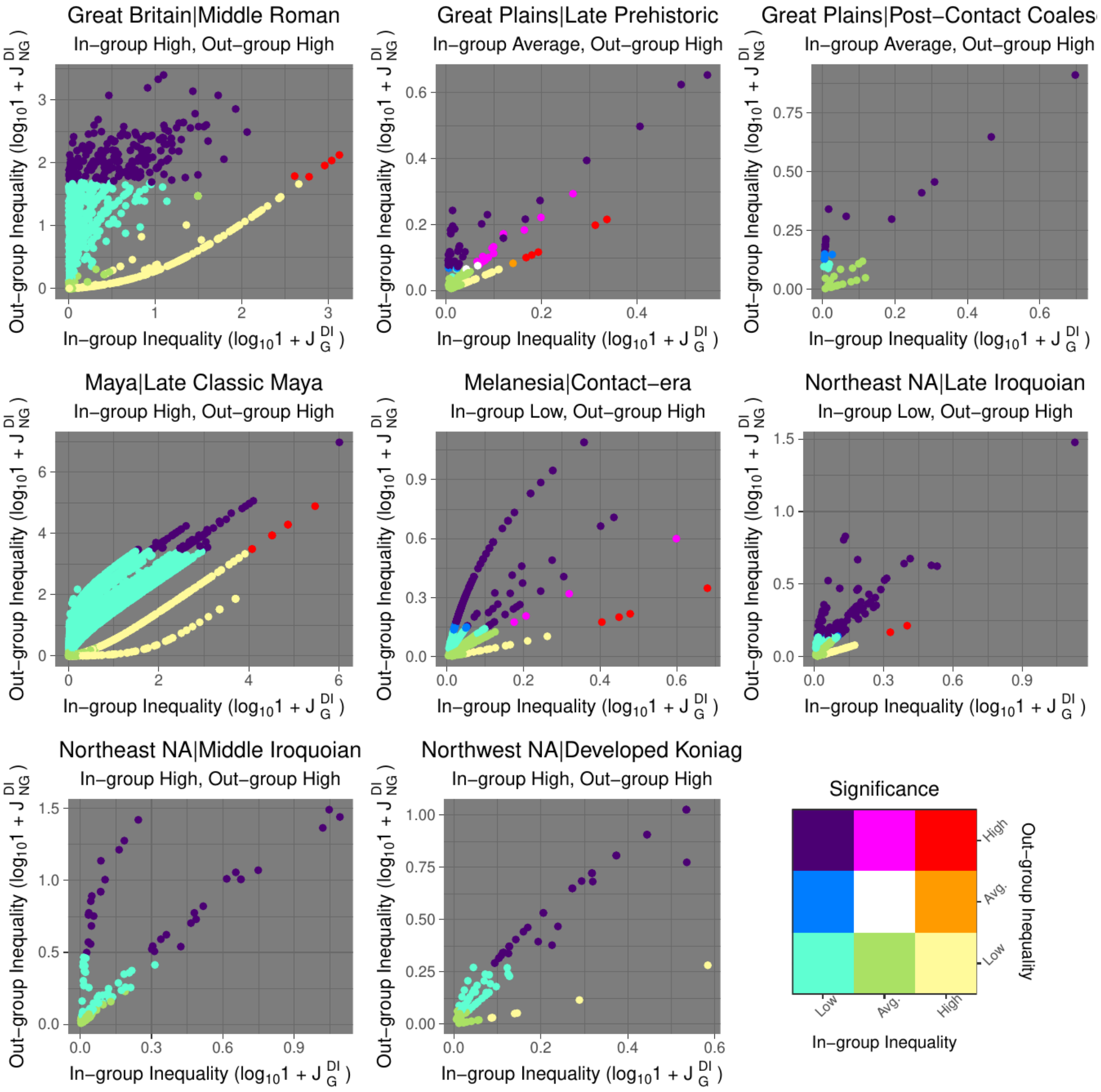

Fig. S7. Residential unit level in-group and out-group inequality per [Region][SitePhase] (3 of 8)

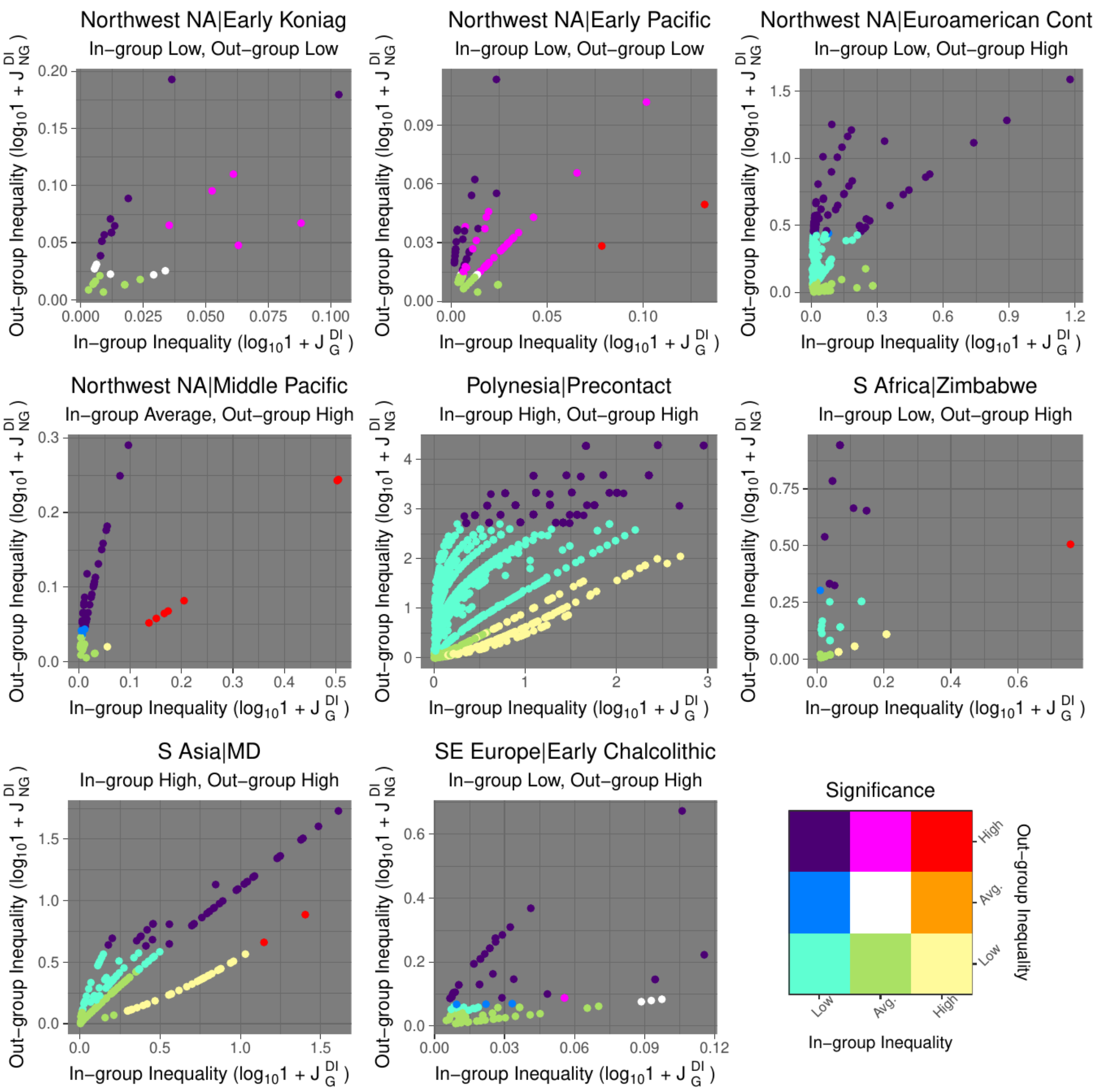

Fig. S8. Residential unit level in-group and out-group inequality per [Region][SitePhase] (4 of 8)

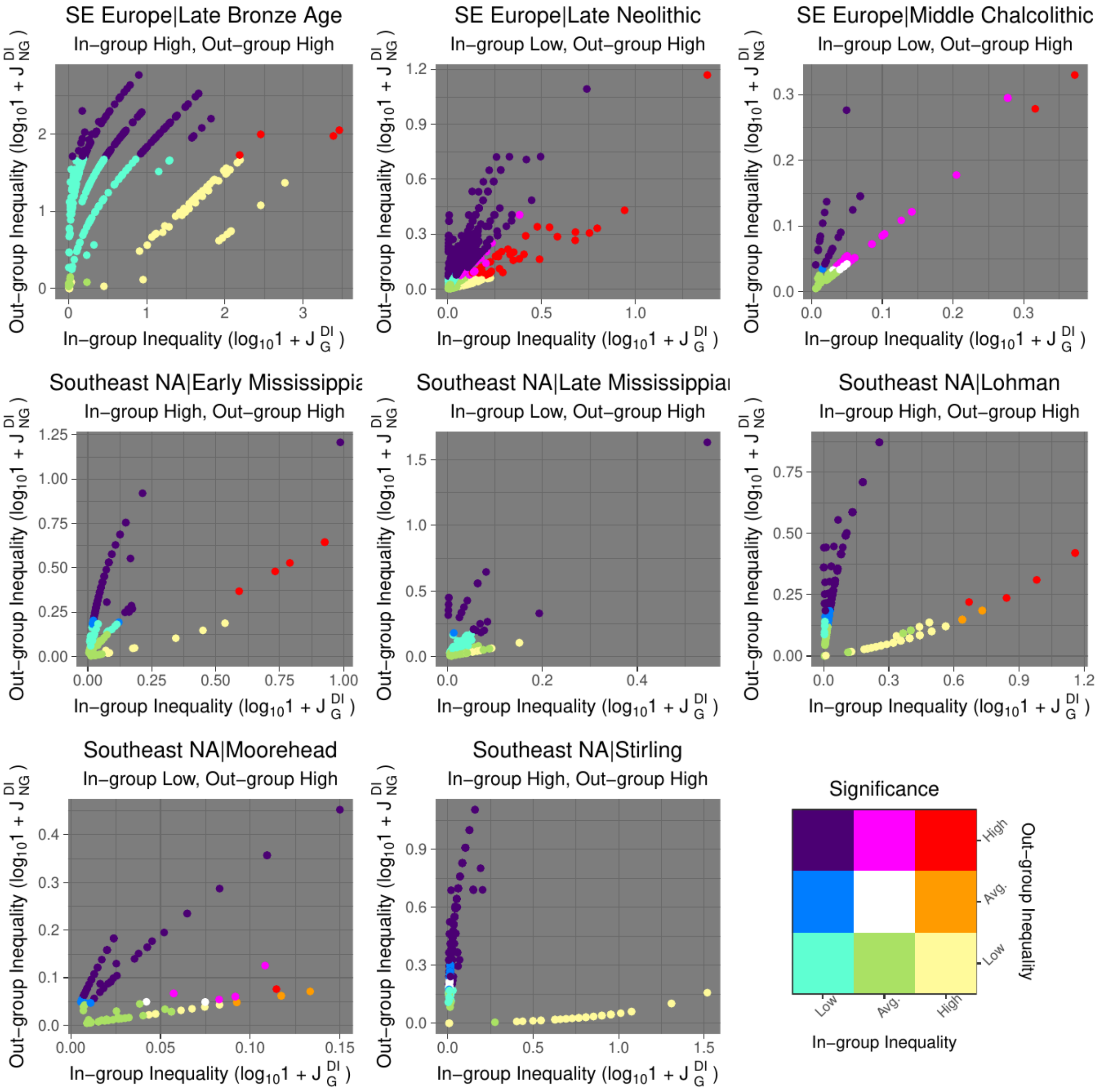

Fig. S9. Residential unit level in-group and out-group inequality per [Region][SitePhase] (5 of 8)

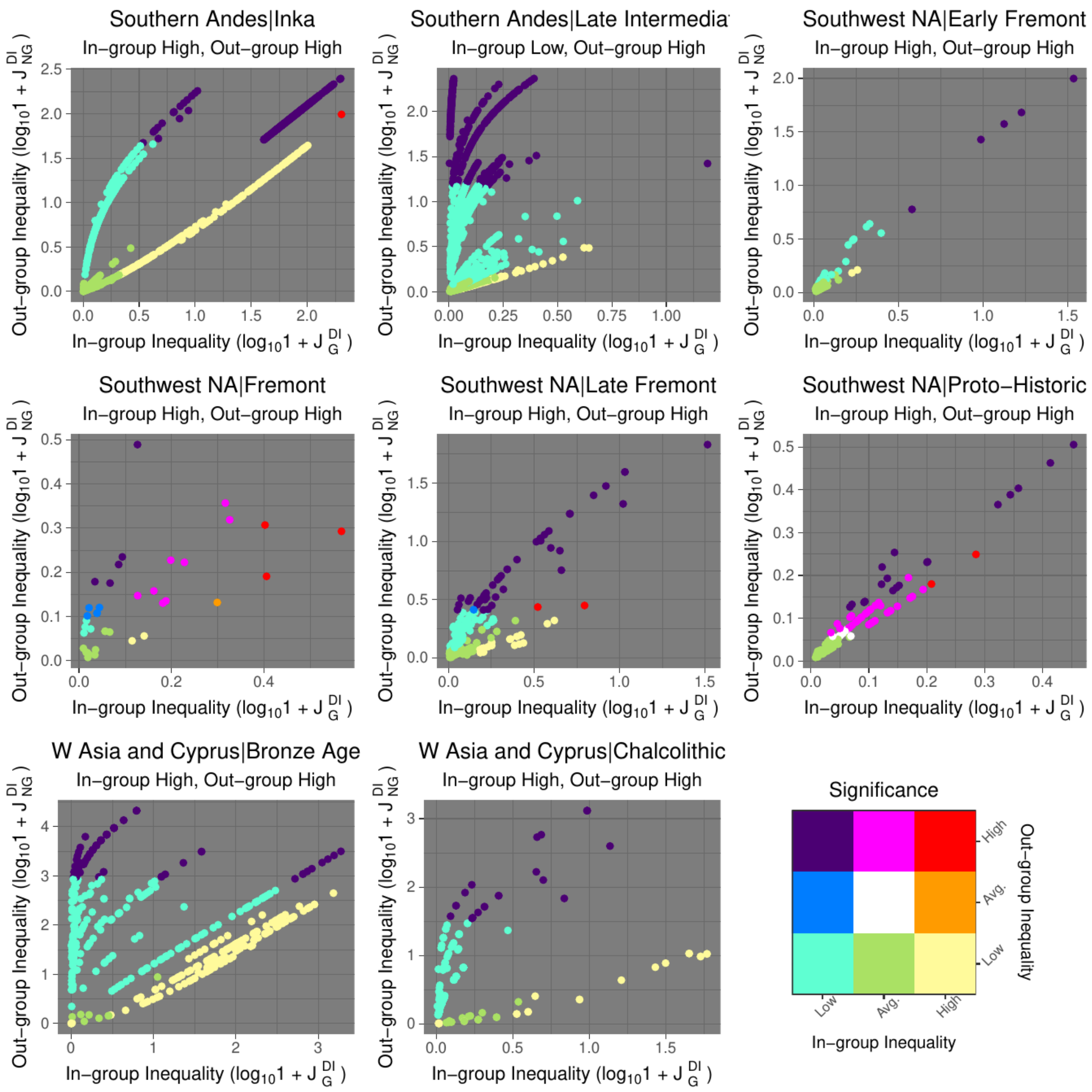

Fig. S10. Residential unit level in-group and out-group inequality per [Region][SitePhase] (6 of 8)

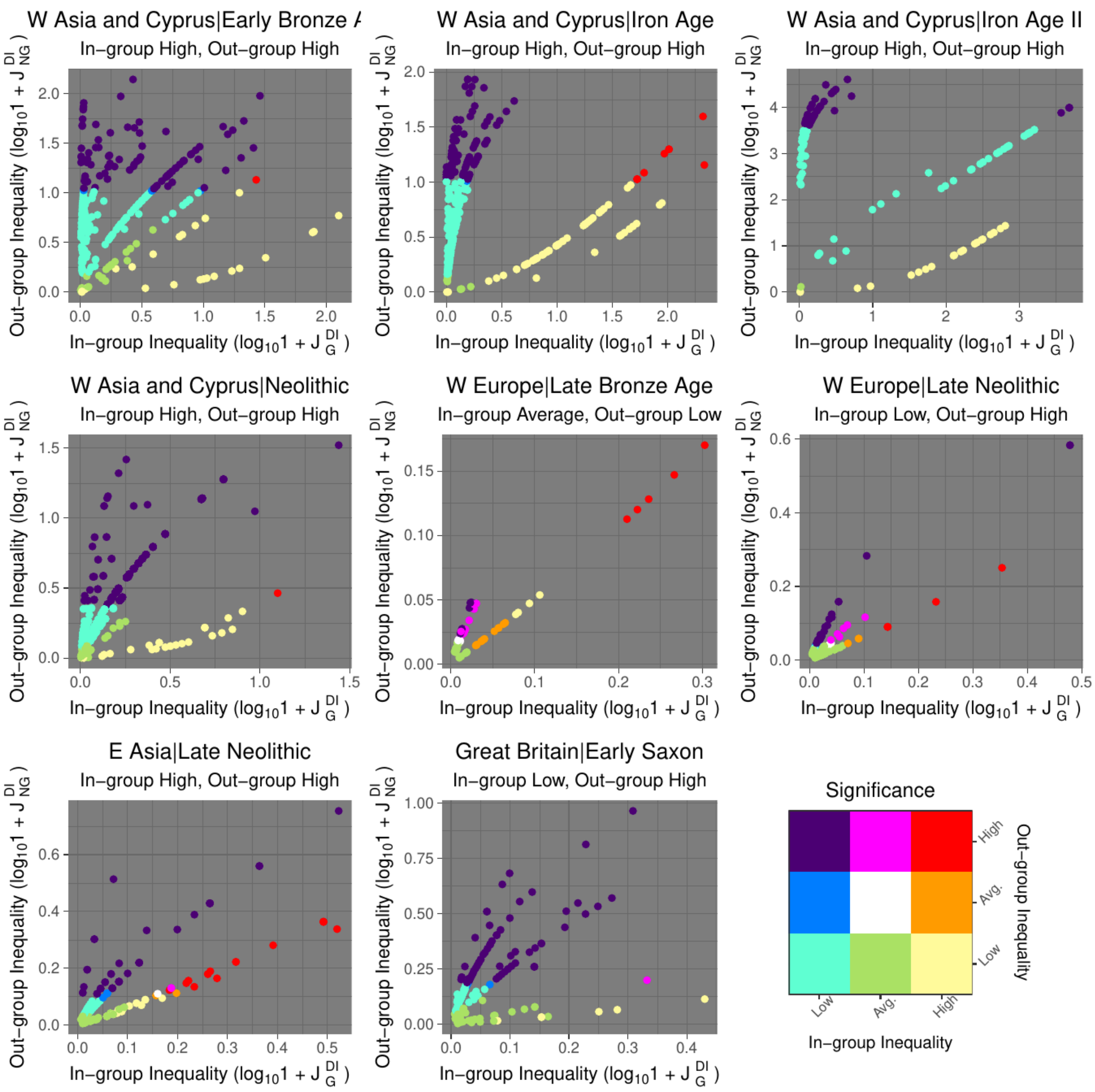

Fig. S11. Residential unit level in-group and out-group inequality per [Region][SitePhase] (7 of 8)

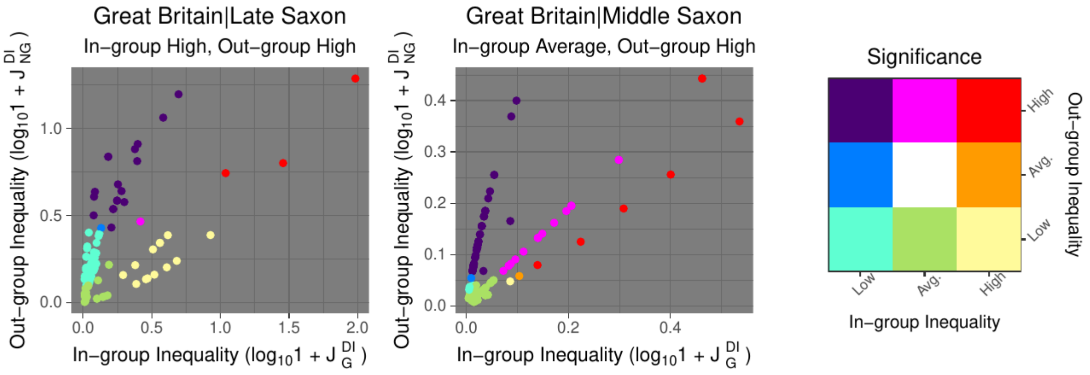

Fig. S12. Residential unit level in-group and out-group inequality per [Region][SitePhase] (8 of 8)

Table S1. Indicators of inequality used in this paper. *x* - value of an observation (in this case the floor area of a domestic residence); *i* and *j* - observations in the dataset; *p(x)* - probability of getting *x* from a sample of the distribution; *c* - value at which the tail begins to behave like a power-law distribution; *J* - standardised deviation from expectation; *G* - group; *NG* - non-group; *n* number of observations; *h* - sites in the dataset.

Metric	Definition	Description
Gini index	$\sum_i \sum_j \frac{\lvert x_i - x_j \rvert}{2\bar{x}}$	Standardised measure of absolute difference. *Strength:* Easy to calculate & intuitively understand meaning. Commonly used in different settings *Weakness:* Poorly sensitive to extreme values.
Inoua index	$\sum_i \sum_j \frac{(x_i - x_j)^2}{2\bar{x}^2}$	Standardised measure of absolute variance. *Strength:* Similar to Gini, but more sensitive to tail. *Weakness:* Difficult to graphically visualize.
α-inequality		Gini index calculated at the smallest scale of aggregation (usually an archaeological site). *Strength:* Can potentially rely on high sampling fraction. *Weakness:* Potentially not representative or partially representative of the societal levels of inequality.
β-inequality	$SD(\alpha)$	Standard deviation of α-inequality within a region. *Strength:* Can provide insights on regional structures of inequality. *Weakness:* Small sampling fraction and potential sampling bias.
γ -inequality		Gini index of the entire region calculated without accounting for lower-level aggregations (i.e. sites). *Strength:* Can rely on larger sample sizes. *Weakness:* Can be biased by specific samples available and confounded by factors such as β-inequality and variation in median observations.
δ-inequality	$\gamma - \bar{\alpha}$	Difference between γ-inequality and average α-inequality. *Strength:* Can provide a measure of discrepancy between typical low-level inequality and regional patterns. *Weakness:* Multiple factors contribute to its value, making it of limited explanatory value.
Scaling factor η	$(\gamma - \alpha_h)/(n_G - n_h)$	The difference between γ-inequality and α-inequality of a given site *h* normalised with the difference in the regional and site-specific sample sizes. *Strength:* Can provide a measure of how the sample size affects the difference in Gini at different scale levels irrespective of other confounding factors (e.g. political and demographic complexity). *Weakness:* Can be biased by limited or non-random sampling.

-*k* exponent	$\frac{\ln p(x)}{\ln (x) + c}$	Power law scaling exponent. *Strength:* Identifies 'rich get richer' dynamics; gives insight to underlying generative processes.*Weakness:* Only applicable for Power law and Pareto distributions. Highly sensitive to biased samples
Local Indicators of Dispersion	$\sum_{i=1}^{n}\sum_{j=1}^{n} J_{Gi}\frac{w_{ij}}{n^2} + \sum_{i=1}^{n}\sum_{j=1}^{n} J_{NGi}(1 - \frac{w_{ij}}{n^2})$	Weighted sum of standardised deviation from expectations by group and non-group members. *Strength:* Can calculate in- and out-group inequality; flexible for different use cases. *Weakness:* Researcher has to identify the appropriate standards and expectations. Sometimes unbounded.
